# The Limitations of the GRE in Predicting Success in Biomedical Graduate School

**DOI:** 10.1371/journal.pone.0166742

**Published:** 2017-01-11

**Authors:** Liane Moneta-Koehler, Abigail M. Brown, Kimberly A. Petrie, Brent J. Evans, Roger Chalkley

**Affiliations:** 1 The Office of Biomedical Research Education & Training, Vanderbilt University School of Medicine, Nashville, Tennessee, United States of America; 2 Department of Leadership, Policy & Organizations, Peabody College, Vanderbilt University, Nashville, Tennessee, United States of America; Northwestern University, UNITED STATES

## Abstract

Historically, admissions committees for biomedical Ph.D. programs have heavily weighed GRE scores when considering applications for admission. The predictive validity of GRE scores on graduate student success is unclear, and there have been no recent investigations specifically on the relationship between general GRE scores and graduate student success in biomedical research. Data from Vanderbilt University Medical School’s biomedical umbrella program were used to test to what extent GRE scores can predict outcomes in graduate school training when controlling for other admissions information. Overall, the GRE did not prove useful in predicating who will graduate with a Ph.D., pass the qualifying exam, have a shorter time to defense, deliver more conference presentations, publish more first author papers, or obtain an individual grant or fellowship. GRE scores were found to be moderate predictors of first semester grades, and weak to moderate predictors of graduate GPA and some elements of a faculty evaluation. These findings suggest admissions committees of biomedical doctoral programs should consider minimizing their reliance on GRE scores to predict the important measures of progress in the program and student productivity.

## Introduction

The goal of biomedical graduate Ph.D. programs is to identify and train students for the purpose of advancing biomedical research. Admissions committees are charged with the task of predicting who will be the best Ph.D. students given somewhat limited information about the applicants’ past performance: undergraduate grade point average (GPA); Graduate Record Exam (GRE) Quantitative, Verbal, and Writing scores; letters of recommendation; and a personal statement. GRE scores are highly influential in the selection process [1,2], yet past research is unclear regarding the ability of GRE scores to predict students’ graduate performance, with some studies showing weak correlations with graduate school grades [3] and some studies showing a more robust impact of GRE scores on student outcomes [4–6].

The Educational Testing Service (ETS), which administers the GRE, advises restrained use of general test scores for admissions and discourages the use of a cutoff score [7]. According to their own studies, the GRE correlates slightly with graduate GPA [8] and does not predict other skills needed to succeed in a variety of graduate programs [9]. It also has been argued that the GRE is a racially and socioeconomically biased test [1] similar to arguments made about the SAT and ACT at the undergraduate level [10]. For example, from 2009–2010, White GRE test takers scores on the Quantitative, Verbal, and Analytical Writing subtests were 18–32% higher than Black test takers [11]. Moreover, students with a low socioeconomic status (SES) perform worse on standardized tests, and exams like the SAT are highly correlated with parental income [12]. Explanations for these differences include student access to academic preparation such as prior schooling or test prep courses [13], stereotype threat [14], and even the inability to pay to retake the $195 test after receiving a low score. Regardless of the reason, certain groups perform worse than others on the exam, and schools that demand high GRE scores for admission may be systematically disadvantaging specific racial and socioeconomic groups. African Americans, Hispanics, Native Americans, and Hawaiian/Pacific Islanders, as well as low SES individuals, are already underrepresented in the biomedical research workforce [15]. A reliance on using the GRE for admission decisions may limit their ability to enter the field.

Biomedical research graduate programs have grown in size significantly over the last ten years [16], and many of these programs emphasize GRE scores for admissions decisions [2]. Few studies focus specifically on the relationship between biomedical Ph.D. student success and GRE scores. A recent study of 57 Puerto Rican biomedical students at Ponce Health Sciences University revealed a shared variance between GRE and months to defense (r^2^ = .24), but no relationship between GRE score and degree completion or fellowship attainment [17]. Another small study of 52 University of California San Francisco (UCSF) biomedical graduate students attempted to show that general GRE scores are not predictive of student success [18], however, as UCSF students address in their critique, a vague definition of success and weak research methods confound the interpretation [19]. The UCSF students conclude that more rigorous studies, such as this one, are needed.

Larger studies of biomedical graduate students have shown shared variance between GRE scores and graduate GPA (r^2^ ranging from .05 to .25), as well as faculty ratings of student performance (r^2^ ranging from .05 to .25), but rely on data that are 19 or more years old and do not account for more recent changes to the exam [4,5,20]. ETS regularly updates questions and changed the GRE in 2002, replacing the Analytical Ability section with the Analytical Writing Assessment section. Newer GRE scores may show different predictions for biomedical Ph.D. student success than they did 19 years ago, and there appears to be no study that examines the individual contribution of the GRE Writing subtest on biomedical doctoral student outcomes. Furthermore, Ph.D. students have changed. Incoming cohorts of students are vastly more diverse [21] and with more robust research experience [22] than in previous years. The revision of the GRE in concert with changes in student populations prompt this focused and updated investigation.

The Vanderbilt Interdisciplinary Graduate Program (IGP) is an umbrella admissions program that started in 1992 and serves the graduate programs in biochemistry, biological sciences, cancer biology, cell and developmental biology, cellular and molecular pathology, chemical and physical biology, human genetics, microbiology and immunology, molecular physiology and biophysics, neurosciences, and pharmacology. Students may apply directly to a biomedical department, however the majority choose to enter through the IGP. IGP students are required to complete a common first semester course, supplemented by electives in the spring semester, and 3–4 rotations in different research laboratories across the 11 different programs and departments. After students complete the first year, they enter a specific degree granting program to continue their studies.

The IGP admissions committee meets weekly during late winter to make admission decisions. It is made up of 13 faculty and a representative of diversity initiatives. GRE Quantitative, Verbal, and Writing scores (maximum scores in the case of multiple tests) are used for admissions decisions, along with undergraduate GPA, letters of recommendation, a personal statement, and, for some, campus visits and interviews. Since the inception of the IGP, there has been no minimum GRE cutoff score, but if an applicant’s GRE scores are low, he or she will have to excel in at least one of the other three application requirements to be competitive for admission.

This study investigates the predictive validity of GRE scores on various quantitative and qualitative measures of success for biomedical Ph.D. students including measures of progress in the program (passing the qualifying exam, graduation with a Ph.D., and time to defense), research productivity (presentation and first author publication rates and obtaining individual grants and fellowships), grades (first semester and overall GPAs), and faculty evaluations of students obtained at the time of thesis defense. Faculty evaluations, while being subjective measures of success, are important for the IGP given that most faculty do not directly select graduate students to enter their labs. Instead the admissions committee selects a cohort of biomedical students that they hope will meet the expectations of their faculty colleagues. Post-graduate career outcomes were excluded from the study, as we are hesitant to categorize one career as more or less successful than another. This, this study focuses solely on measures of success up to and including graduation.

We explore the importance of the GRE General Test in the biomedical field using a large and up to date dataset. This study covers hundreds of students from 11 departments and programs and looks at a wider range of outcomes and control variables than prior studies. Such an up-to-date, comprehensive evaluation of the use of the GRE in evaluating prospective biomedical graduate students is important to ensure that the admissions process aligns with the goals of the institution and to determine whether a GRE requirement for graduate school admission is worth the inherent biases that the test might bring into the admissions process.

## Methods

Data were collected on 683 students who matriculated into the Vanderbilt University IGP from 2003 to 2011, a time period in which reliable GRE scores are available. Over 80% of students have had time to complete the program. GRE Quantitative, Verbal, and Analytical Writing scores were used to test the hypothesis that they could predict several measures of graduate school performance, including (1) graduation with a Ph.D., (2) passing the qualifying exam, (3) time to Ph.D. defense, (4) number of presentations at national or international meetings at time of defense, (5) number of first author peer-reviewed publications at time of defense, (6) obtaining an individual grant or fellowship, (7) performance in the first semester coursework, (8) cumulative graduate GPA, and (9) final assessment of the competence of the student as a scientist as evaluated by the research mentor. In order to determine the independent contributions of GRE scores on outcome measures, additional admissions criteria were included in the analyses as controls: undergraduate GPA, undergraduate institution selectivity, whether a student has a prior advanced degree, underrepresented minority status, international student status, and gender. Details on variables are described below. The research was approved by Vanderbilt University IRB (#151678). Consent was not given as data were analyzed anonymously.

### Independent Variables

Students submitted their GRE scores as part of their application to the IGP. If multiple scores were submitted, superscores (the highest score on each subtest) were used for admissions decisions and for this study. Students also submitted their undergraduate GPAs, undergraduate institutions, prior advanced degree information, minority status, international student status, and gender on their IGP applications. Undergraduate institution selectivity from the 2007–2008 year, the median admissions year for the sample, was acquired from The Integrated Postsecondary Education Data System [23]. Selectivity is calculated by dividing the number of admissions offers by the number of applicants. Lower numbers are associated with more selective schools. Prior advanced degrees include master’s degrees, medical degrees and pharmacy degrees. Students are considered to have underrepresented minority status if they are underrepresented in science as defined by the National Institutes of Health. Minority status specifically denotes individuals from certain racial and ethnic groups (African Americans, Hispanic Americans, Native Americans, Alaskan Natives, Hawaiian Natives, and natives of the U.S. Pacific Islands), individuals with disabilities, and individuals from disadvantaged backgrounds. Students holding temporary visas are categorized as international students. Only international students with undergraduate degrees from U.S. schools were included in the study (see Results).

### Measures of Student Progress through the Ph.D. Program

Shortly after the second year of study, students took a pass/fail qualifying exam to be admitted for Ph.D. candidacy. Subsequently, students spent the remainder of their time in the program on their dissertation research projects. After successful completion of their dissertation research, students then defended their dissertation and graduated with a Ph.D. Some students withdrew from the program leaving with no degree, while others left with a terminal Masters degree. Time to successful Ph.D. defense was calculated by subtracting a student’s matriculation date from his or her defense date and dividing by 365.25 days. Data includes all students who defended their dissertations before May 2016. The current sample of students trained in over 200 different laboratories, which precludes using mentor controls due to the small number of students in each lab.

### Research Productivity

Within two weeks after the dissertation defense, Ph.D. students were invited to complete a voluntary 117-question exit survey. The exit survey covers a wide variety of topics including the number of first-author peer-reviewed scientific papers (published or in press), the number of scientific presentations (poster or podium presentation) given at national or international meetings and conferences, and if they received an individual grant or fellowship while enrolled in the Ph.D. program. Each respondent was limited to a maximum of 12 presentations, a response given by one student in the sample. Only competitive grants and fellowships were considered. Sixty-four percent of the grants and fellowships were supported by federal sources, such as the National Institutes of Health and the National Science Foundation, while the remaining 36% percent were supported by private organizations, such as the American Heart Association. Eleven percent of all awards promote diversity in research. The exit survey began in January, 2007 and has a response rate of over 90%.

### Grades

All students entering the IGP took one semester of intensive core coursework, intended to teach the fundamentals of biomedical research, critical thinking, and how to gain information from the scientific literature. Student received a grade out of 100%. In the spring semester of the IGP year, students took elective courses. At the end of the IGP year, students selected a training program in one of eleven participating departments or programs and completed an additional year of didactic course work and initiated their thesis requirements. The graduate GPA includes all didactic course grades from the first two years of study.

### Faculty Evaluation of Student Immediately After Defense

Within one year of the student’s dissertation defense, the thesis mentor completed a final evaluation of the student. The mentor was asked to rate the student on a scale from one (best possible score) to five (worst possible score) in ten different categories: (1) ability to handle the classwork needed for success in the Ph.D. program, (2) drive and determination, (3) creativity and imagination in terms of experimental design and interpretation, (4) technical ability, (5) keeping up with the literature, (6) output (i.e. translating observations into a presentable paper), (7) ability to write creatively, (8) leadership in the lab and department, (9) trajectory, and (10) overall assessment as a productive scientist. This mentor evaluation began in July, 2007 and has a response rate of over 65%.

## Results

Table 1 shows the descriptive statistics for each of the independent and dependent variables. To control for changes in the sample, analyses were performed on the 495 students showing values for all of the independent variables. See S1 Supporting Information for details on how this group differs from the students for whom we do not have complete data. Within the sample of 495 students, not all students have data for each dependent variable. 19% of students left the program with a Master’s or no degree. Moreover, given that the students took an average of 5.67 years to defend, 17% of students were still active in the program, further reducing the mean number of students that graduated with a Ph.D. Of those that attained a Ph.D., 91% completed the survey asking about presentations, publications, and grants, and 65% received evaluations from their faculty mentors.

**Table 1:** Summary Statistics for each of the Independent and Dependent Variables.

Independent Variable	N	Mean or Proportion^+^	SD
GRE Scores			
GRE Quantitative	495	693.35	67.34
GRE Verbal	495	554.26	84.82
GRE Analytical Writing	495	4.62	0.67
Undergraduate GPA	495	3.54	0.32
Undergraduate Institution Selectivity	495	59.44	20.02
Proportion with Prior Advanced Degree	495	0.05	0.21
Proportion with Underrepresented Minority Status	495	0.12	0.33
Proportion International Students	495	0.05	0.21
Proportion Female	495	0.59	0.49
Dependent Variable			
Proportion Graduated with a Ph.D.	495	0.64	0.48
Proportion Passed Qualifying Exam	495	0.88	0.32
Time to Defense (years)	318	5.67	0.98
Presentation Count	271	4.06	2.32
First Author Publication Count	271	1.79	1.10
Proportion with Individual Grant or Fellowship	271	0.38	8.36
First Semester Grade	488	79.73	0.90
Graduate GPA	492	3.66	0.27
Faculty Evaluation			
Ability to Handle Classwork	210	1.79	0.84
Drive	210	1.98	1.02
Creativity with Experimental Design	210	2.22	0.99
Technical Ability	210	1.85	0.88
Keeping up with Literature	210	2.15	0.96
Output	210	2.11	1.02
Writing	210	2.31	1.03
Leadership	210	2.04	1.06
Trajectory	210	2.09	0.99
Overall Assessment	210	2.09	1.00

^+^Means are reported for continuous variables, and proportions are reported for binary variables.

**Fig 1:**
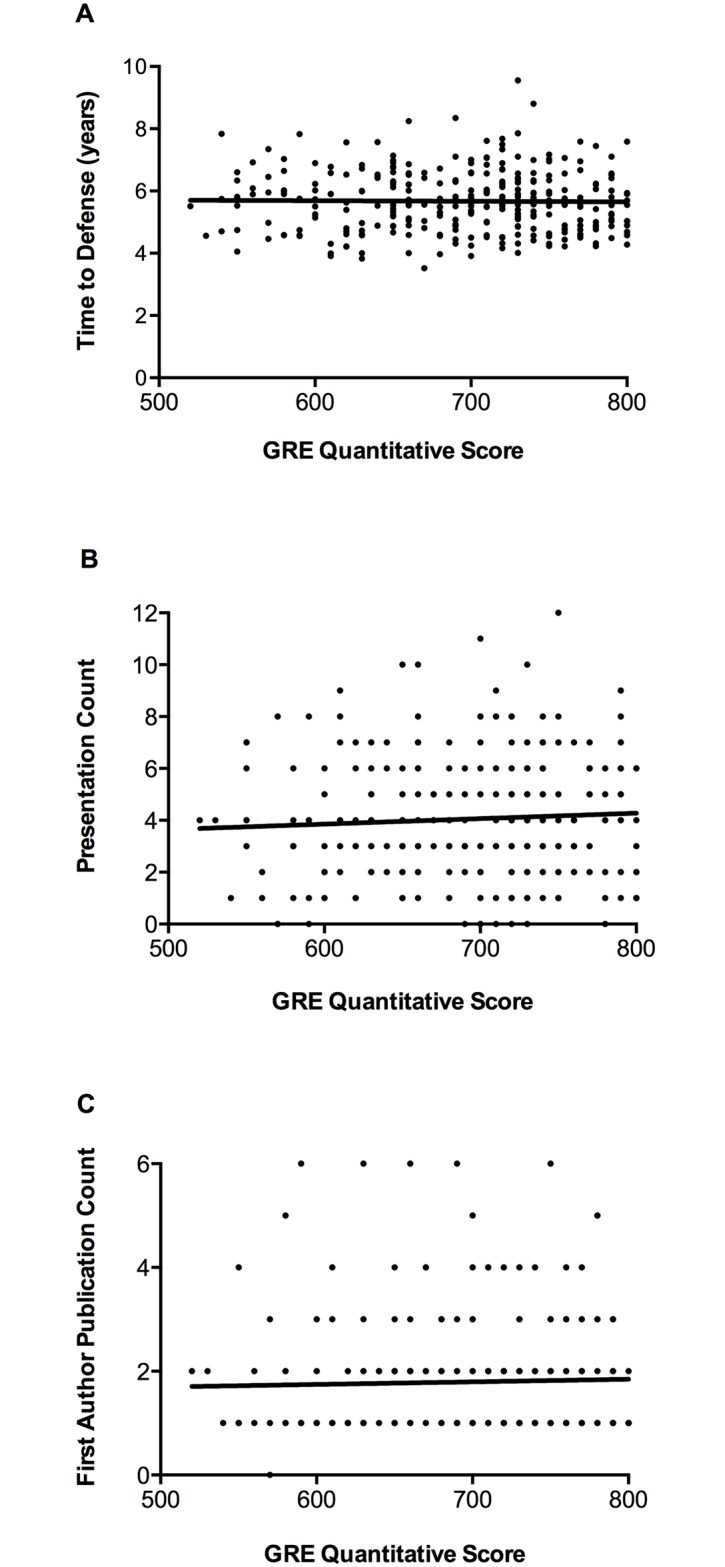
Correlations between GRE Quantitative scores and continuous measures of student progress and productivity. Scatterplots of GRE Quantitative scores and (A) Time to Defense regression coefficient = 0.00, p = .83, R^2^ = 0.00, (B) Presentation Count regression coefficient = 0.00, p = .32, R^2^ = 0.00), and (C) First Author Publication Count (regression coefficient = 0.00, p = .62, R^2^ = 0.00). GRE Quantitative scores do not correlate with these measures of success.

A visual examination of the relationship between GRE Quantitative scores and the continuous measures of progress in the program and research productivity revealed no effect (Fig 1). GRE Quantitative scores did not significantly correlate with Time to Defense (regression coefficient = 0.00, p = .83, R^2^ = 0.00), Presentation Count (regression coefficient = 0.00, p = .32, R^2^ = 0.00), or First Author Publication Count (regression coefficient = 0.00, p = .62, R^2^ = 0.00, see Fig 1). Similar results were found with GRE Verbal and Writing scores. Given that admissions committees do not base decisions on single measures like GRE Quantitative scores and instead look at a collection of admissions criteria, we have examined the influence of multiple measures as they pertain to graduate student success.

Following a line of research that examines predictive validity of test scores, in order to evaluate the influences of each independent variable in the presence of the other admission criteria, linear regression analyses were used [24,25]. Admission cohort was included as a fixed effect to account for systematic changes that occur over time. We first looked at the influence of GRE scores and other admissions criteria on measures of progress in the program, defined as Passing the Qualifying Exam, Graduation with a Ph.D., and Time to Defense. We then investigated measures of productivity (Presentation Count, First Author Publication Count, and Obtaining an Individual Grant or Fellowship), grades (First Semester GPA and Graduate GPA), and faculty evaluations.

**Fig 2:**
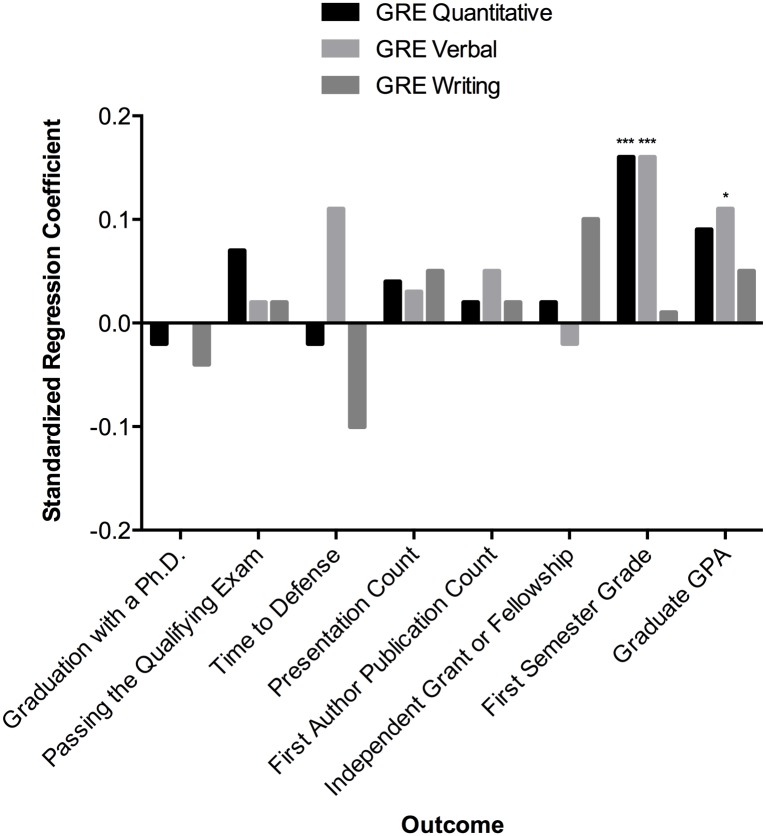
The predictive power of GRE scores on different measures of student success. Standardized regression coefficients reported after controlling for other admissions criteria. Cohort fixed effects are included for each model. Coefficients of zero appear as missing bars. *p < .05. **p < .01. ***p < .001.

Fig 2 provides an overview of each GRE subtest’s relationship with eight different measures of student success after controlling for other admission criteria. Standardized regression coefficients reflect effect sizes such that, for example, one standard deviation change in GRE Quantitative is associated with a 0.16 standard deviation change in First Semester GPA. Standardized correlation coefficients were used in order to make comparisons across variables. Later analyses left binary variables unstandardized. We can see that GRE Verbal scores were a better predictor of First Semester Grades than Graduate GPA due to the higher standardized regression coefficient for First Semester Grades. In sum, GRE scores showed some validity in predicting classroom performance but not progress in the program or research productivity.

### GRE Scores Do Not Predict Progress in the Program

The results collected in Table 2 allowed us to see the effect of GRE scores upon an outcome variable collected during graduate training, in this case graduation with a Ph.D. Continuous independent variables (GRE Quantitative, GRE Verbal, GRE Writing, Undergraduate GPA, and Undergraduate Institution Selectivity) were standardized before entering the regression, whereas binary independent variables (Prior Advanced Degree, Underrepresented Minority, International, and Female) were not standardized and are shaded in gray to indicate that they are unstandardized regression coefficients. We used linear probability models for the binary dependent variables, so the coefficients should be interpreted as a change in the probability of the event happening (i.e. graduating). The table is constructed to display first the effect of a single variable, GRE Quantitative, on Graduation with a Ph.D. (Column (1)). The simple bivariate regression between GRE Quantitative and Graduation with a Ph.D. revealed no influence of GRE Quantitative scores. Moving rightward, when GRE Verbal and Writing scores were added to the model (Column (3)), none were shown to predict Graduation with a Ph.D. The rightmost column (Column (9)) is especially informative as it shows the independent contribution of each GRE subtest after controlling for all the other observed admissions variables. Again, none of the GRE subtests predicted graduating with a Ph.D. Undergraduate GPA significantly predicted Gradation with a Ph.D., such that one standard deviation increase in Undergraduate GPA was associated with a 0.05 increase in the probability of attaining a Ph.D. Note that one standard deviation of Undergraduate GPA in the sample was 0.32 on a 4 point scale (Table 1). Underrepresented Minority status also predicted Graduation with a Ph.D., such that Underrepresented Minority students had a 0.13 decrease in the probability of attaining a Ph.D relative to non-minority students. The full model accounted for 29% of the variance in Graduation with a Ph.D. (see Adjusted R^2^ in Table 2), most of which was driven by the inclusion of cohort fixed effects which control for a host of unobserved factors that were consistent with cohort. We chose to present linear probability models because their coefficients are directly interpretable as changes in the probability of graduating; however, logit models showed the same sign and significance and thus were qualitatively similar to the linear regression results.

**Table 2:** The predictive power of GRE scores and other admissions criteria on Graduation with a Ph.D.

	(1)	(2)	(3)	(4)	(5)	(6)	(7)	(8)	(9)
GRE Quantitative	0.00	0.00	0.00	0.00	-0.01	0.00	-0.01	-0.01	-0.01
	[-0.04, 0.03]	[-0.04, 0.04]	[-0.04, 0.04]	[-0.04, 0.04]	[-0.05, 0.03]	[-0.04, 0.04]	[-0.05, 0.03]	[-0.05, 0.03]	[-0.05, 0.03]
GRE Verbal		0.00	0.01	0.01	0.00	0.00	0.00	0.00	0.00
		[-0.04, 0.05]	[-0.03, 0.05]	[-0.04, 0.05]	[-0.04, 0.04]	[-0.04, 0.04]	[-0.05, 0.04]	[-0.04, 0.04]	[-0.04, 0.04]
GRE Writing			-0.01	-0.01	-0.02	-0.02	-0.02	-0.02	-0.02
			[-0.05, 0.03]	[-0.05, 0.03]	[-0.06, 0.02]	[-0.06, 0.02]	[-0.06, 0.02]	[-0.06, 0.02]	[-0.06, 0.02]
Undergraduate GPA				0.04*	0.05*	0.05**	0.05**	0.05*	0.05*
				[0.00, 0.08]	[0.01, 0.09]	[0.01, 0.09]	[0.01, 0.09]	[0.01, 0.09]	[0.01, 0.09]
Undergraduate Inst. Selectivity					-0.02	-0.03	-0.03	-0.03	-0.03
					[-0.06, 0.02]	[-0.06, 0.01]	[-0.07, 0.01]	[-0.07, 0.01]	[-0.06, 0.02]
Prior Advanced Degree						0.13	0.14	0.13	0.13
						[-0.04, 0.31]	[-0.04, 0.31]	[-0.05, 0.31]	[-0.05, 0.31]
Underrepresented Minority							-0.12*	-0.13*	-0.13*
							[-0.24, 0.00]	[-0.25, -0.01]	[-0.24, -0.01]
International								0.08	0.08
								[-0.10, 0.26]	[-0.10, 0.26]
Female									0.02
									[-0.05, 0.10]
Adjusted R-Squared	0.28	0.28	0.28	0.29	0.29	0.29	0.29	0.29	0.29
Observations	495	495	495	495	495	495	495	495	495

Regression coefficients and 95% confidence intervals are reported from linear regressions of Graduation with a Ph.D. on GRE Quantitative (Column (1)), the addition of the other GRE subtests (Columns (2) and (3)) and the addition of other admissions variables (Columns (4)-(9)). Continuous variables were standardized prior to entering the models. Binary variables are shaded gray. Cohort fixed effects are included for each model. Adjusted R-squared is presented for each model.

*p < .05.

**p < .01.

***p < .001.

Similar linear regression analyses were run to predict a student’s passing the Qualifying Exam and Time to Defense. The results are in Tables A and B in S1 Supporting Information. Continuous independent and dependent variables were standardized before entering the regressions. When all admission criteria are entered into the model, no variable predicted a student’s likelihood of passing the Qualifying Exam or Time to Defense.

### GRE Scores Do Not Predict Research Productivity

Linear regression analyses were used to compare GRE scores to quantitative measures of research productivity. These measures include Presentation Count (Table C in S1 Supporting Information), First Author Publication Count (Table D in S1 Supporting Information), and obtaining an Individual Grant or Fellowship (Table E in S1 Supporting Information). Continuous independent and dependent variables were standardized before entering the regressions. When all admission criteria were included in the models, none of the GRE subtests predicted the above dependent variables. Minority Status was the only significant predictor of obtaining an Individual Grant or Fellowship, and no variables significantly predicted Presentation Count or First Author Publication Count. GRE scores and most standard objective measures for admissions did not predict measures of student productivity.

### GRE Scores Moderately Predict Grades

Linear regressions were run to examine student classroom performance, starting with First Semester Grades (Table 3). For this continuous outcome, the variable was standardized, as were all of the continuous independent variables such that regression coefficients can be interpreted as effect sizes. The shaded, binary independent variables remained unstandardized. GRE Quantitative scores moderately predicted First Semester GPA (Column (1)). A one standard deviation increase in GRE Quantitative was associated with a 0.29 standard deviation increase in First Semester Grades (Column (1)). When GRE Verbal scores were added (Column (2)), the model accounted for an additional 4% of the variance in First Semester Grades. GRE Writing scores did not predict First Semester Grades. GRE Quantitative and Verbal continued to predict First Semester Grades after controlling for other factors (Column (9)), although the magnitude of the relationship was attenuated with the inclusion of other predictors, given their overlapping influence on grades. Undergraduate GPA, Admission Rate, and Underrepresented Minority status also predicted First Semester Grades, with Undergraduate GPA having a higher coefficient than the GRE subtests. Undergraduate Institution Selectivity negatively contributed to First Semester Grades. Since competitive schools have lower selectivity scores, higher selectivity represents less competitive schools and predicted lower First Semester Grades. Underrepresented Minority status was associated with a 0.35 standard deviation decrease in grades. When all admissions variables were included, the model accounted for 40% of the variance in First Semester Grades, and was strongly driven by the cohort fixed effect.

**Table 3:** The predictive power of GRE scores and other admissions criteria on First Semester Grades.

	(1)	(2)	(3)	(4)	(5)	(6)	(7)	(8)	(9)
GRE Quantitative	0.29***	0.20***	0.20***	0.18***	0.18***	0.18***	0.16***	0.16***	0.16***
	[0.22, 0.37]	[0.12, 0.28]	[0.12, 0.28]	[0.11, 0.26]	[0.10, 0.26]	[0.11, 0.26]	[0.08, 0.24]	[0.08, 0.24]	[0.08, 0.24]
GRE Verbal		0.22***	0.21***	0.19***	0.18***	0.18***	0.17***	0.16***	0.16***
		[0.14, 0.30]	[0.12, 0.29]	[0.11, 0.27]	[0.10, 0.27]	[0.10, 0.26]	[0.08, 0.25]	[0.08, 0.24]	[0.08, 0.24]
GRE Writing			0.04	0.03	0.02	0.02	0.01	0.01	0.01
			[-0.04, 0.12]	[-0.05, 0.11]	[-0.06, 0.10]	[-0.06, 0.10]	[-0.06, 0.09]	[-0.07, 0.09]	[-0.07, 0.09]
Undergraduate GPA				0.25***	0.27***	0.27***	0.27***	0.27***	0.28***
				[0.17, 0.32]	[0.19, 0.34]	[0.20, 0.35]	[0.20, 0.35]	[0.20, 0.35]	[0.20, 0.35]
Undergraduate Inst. Selectivity					-0.07	-0.07	-0.08*	-0.08*	-0.08*
					[-0.14, 0.01]	[-0.15, 0.01]	[-0.15, 0.00]	[-0.16, -0.01]	[-0.16, -0.01]
Prior Advanced Degree						0.23	0.24	0.24	0.24
						[-0.11, 0.57]	[-0.10, 0.57]	[-0.09, 0.58]	[-0.09, 0.58]
Underrepresented Minority							-0.37**	-0.35**	-0.35**
							[-0.59, -0.14]	[-0.58, -0.13]	[-0.58, -0.13]
International								-0.13	-0.13
								[-0.48, 0.21]	[-0.48, 0.21]
Female									-0.02
									[-0.17, 0.12]
Adjusted R-Squared	0.29	0.33	0.33	0.39	0.39	0.39	0.40	0.40	0.40
Observations	488	488	488	488	488	488	488	488	488

Standardized regression coefficients and 95% confidence intervals are reported for continuous variables. Coefficients on the binary variables are shaded gray and report the standard deviation change in the outcome by moving from 0 to 1 on the binary variable. Cohort fixed effects are included for each model. Adjusted R-squared is presented for each model.

*p < .05.

**p < .01.

***p < .001.

Students took didactic courses for their first two years of graduate school, and grades from those courses comprise the Graduate GPA (examined in Table 4). Column (2) shows that the model with the GRE Quantitative and Verbal subtest predicted 8% of the variance of Graduate GPA and each independently made significant contributions to the prediction. However, Undergraduate GPA, Undergraduate Institution Selectivity, and Underrepresented Minority status were also related to Graduate GPA (Column (9)) and when included in the model, GRE Verbal was the only GRE subtest to predict Graduate GPA, and to a lesser degree than Undergraduate GPA. Quantitative and GRE Writing scores did not predict Graduate GPA when controlling for other admissions variables. When all variables were analyzed the model, including the cohort fixed effect, predicted 17% of the variance in Graduate GPA.

**Table 4:** The predictive power of GRE scores and other admissions criteria on Graduate GPA.

	(1)	(2)	(3)	(4)	(5)	(6)	(7)	(8)	(9)
GRE Quantitative	0.22***	0.14**	0.13**	0.12*	0.11*	0.12*	0.07	0.08	0.09
	[0.14, 0.31]	[0.05, 0.24]	[0.04, 0.23]	[0.03, 0.21]	[0.02, 0.20]	[0.02, 0.21]	[-0.02, 0.17]	[-0.02, 0.17]	[-0.01, 0.18]
GRE Verbal		0.20***	0.17***	0.16**	0.15**	0.14**	0.12*	0.11*	0.11*
		[0.10, 0.29]	[0.07, 0.27]	[0.06, 0.25]	[0.05, 0.24]	[0.05, 0.24]	[0.02, 0.21]	[0.02, 0.21]	[0.02, 0.21]
GRE Writing			0.09	0.08	0.07	0.07	0.05	0.05	0.05
			[-0.01, 0.18]	[-0.01, 0.17]	[-0.02, 0.16]	[-0.03, 0.16]	[-0.04, 0.14]	[-0.04, 0.14]	[-0.04, 0.14]
Undergraduate GPA				0.22***	0.25***	0.26***	0.25***	0.26***	0.25***
				[0.14, 0.31]	[0.16, 0.34]	[0.17, 0.35]	[0.16, 0.34]	[0.17, 0.34]	[0.16, 0.34]
Undergraduate Inst. Selectivity					-0.09	-0.09	-0.10*	-0.11*	-0.11*
					[-0.18, 0.00]	[-0.18, 0.00]	[-0.19, -0.02]	[-0.20, -0.02]	[-0.20, -0.02]
Prior Advanced Degree						0.22	0.23	0.24	0.24
						[-0.19, 0.62*]*	[-0.17, 0.63]	[-0.16, 0.64]	[-0.16, 0.64]
Underrepresented Minority							-0.66***	-0.65***	-0.65***
							[-0.93, -0.40]	[-0.92, -0.38]	[-0.92, -0.38]
International								-0.11	-0.11
								[-0.52, 0.29]	[-0.52, 0.30]
Female									0.08
									[-0.09, 0.25]
Adjusted R-Squared	0.05	0.08	0.08	0.13	0.13	0.13	0.17	0.17	0.17
Observations	492	492	492	492	492	492	492	492	492

Standardized regression coefficients and 95% confidence intervals are reported for continuous variables. Coefficients on the binary variables are shaded gray and report the standard deviation change in the outcome by moving from 0 to 1 on the binary variable. Cohort fixed effects are included for each model. Adjusted R-squared is presented for each model.

*p < .05.

**p < .01.

***p < .001.

### GRE Scores Predict Some Responses to Mentor Evaluations after Defense

After a student defended his or her dissertation, the faculty mentor completed a 10-question evaluation. Linear regression analyses were run to examine the influence of each admissions variable on answers to individual questions from the faculty evaluations (Table 5). Each column represents a different faculty measured outcome, and only the full models with all of the independent variables are presented. Faculty evaluation ratings were standardized before entering the models. Because a faculty evaluation rating of one is the highest score and five is the lowest, a negative regression coefficient indicates that a variable predicts good graduate school performance. Higher GRE Verbal scores predicted better faculty evaluations of a student’s ability to handle classwork, keep up with the literature, and write creatively. Undergraduate GPA also contributed to faculty evaluations of a student’s ability to handle classwork and write creatively. GRE Writing scores were related to leadership in the lab or department, and Undergraduate Selectivity predicted classwork, creativity in terms of experimental design, and the overall assessment. Having a prior advanced degree had a reverse relationship with faculty evaluations of technical ability and leadership, and International student status had a reverse relationship with evaluations of ability to keep up with the literature. There were no consistent patterns across the different faculty ratings and most admission criteria, making it hard to predict faculty evaluations with information available during the admissions process.

**Table 5:** The predictive power of all admissions criteria on each response from the Mentor Evaluation After Defense.

	Classwork	Drive	Experimental Design	Technical Ability	Reading Literature	Output	Writing	Leadership	Trajectory	Overall
GRE Quantitative	-0.06	0.02	-0.07	-0.09	-0.01	-0.01	-0.04	0.03	0.04	0.00
	[-0.22, 0.09]	[-0.15, 0.19]	[-0.24, 0.10]	[-0.25, 0.07]	[-0.18, 0.15]	[-0.18, 0.15]	[-0.20, 0.12]	[-0.14, 0.19]	[-0.13, 0.21]	[-0.17, 0.16]
GRE Verbal	-0.29***	0.10	-0.01	0.13	-0.17*	-0.04	-0.17*	0.00	0.02	0.04
	[-0.43, -0.14]	[-0.06, 0.26]	[-0.17, 0.15]	[-0.02, 0.29]	[-0.32, -0.01]	[-0.20, 0.12]	[-0.32, -0.01]	[-0.15, 0.16]	[-0.14, 0.18]	[-0.12, 0.20]
GRE Writing	-0.03	-0.06	0.07	-0.03	0.01	-0.12	-0.03	-0.17*	-0.08	-0.05
	[-0.18, 0.11]	[-0.22, 0.10]	[-0.09, 0.23]	[-0.18, 0.12]	[-0.15, 0.16]	[-0.28, 0.04]	[-0.18, 0.13]	[-0.33, 0.01]	[-0.23, 0.08]	[-0.21, 0.10]
Undergraduate GPA	-0.15*	0.02	-0.04	-0.10	-0.10	-0.03	-0.16*	-0.07	-0.04	-0.09
	[-0.29, -0.01]	[-0.13, 0.17]	[-0.19, 0.10]	[-0.24, 0.04]	[-0.25, 0.04]	[-0.18, 0.12]	[-0.30, -0.01]	[-0.22, -0.08]	[-0.19, 0.11]	[-0.24, 0.06]
Undergraduate Inst. Selectivity	0.15*	0.01	0.18*	0.11	0.10	0.10	0.10	0.04	0.14	0.17*
	[0.01, 0.29]	[-0.14, 0.17]	[0.03, 0.33]	[-0.04, 0.26]	[-0.05, 0.25]	[-0.05, 0.25]	[-0.04, 0.25]	[-0.11, 0.19]	[-0.02, 0.29]	[0.02, 0.33]
Prior Advanced Degree	0.26	0.47	0.21	0.73*	0.16	0.21	0.25	0.78*	0.49	0.60
	[-0.36, 0.88]	[-0.21, 1.15]	[-0.46, 0.88]	[0.07, 1.38]	[-0.50, 0.83]	[-0.46, 0.88]	[-0.39, 0.89]	[0.11, 1.44]	[-0.18, 1.16]	[-0.07, 1.26]
Underrepresented Minority	0.11	0.04	0.36	0.40	0.00	0.03	0.40	0.44	0.24	0.21
	[-0.39, 0.62]	[-0.52, 0.59]	[-0.19, 0.90]	[-0.13, 0.93]	[-0.54, 0.54]	[-0.52, 0.57]	[-0.13, 0.92]	[-0.11, 0.98]	[-0.31, 0.78]	[-0.34, 0.75]
International	0.44	-0.13	0.22	0.40	0.70*	-0.06	0.21	-0.19	-0.05	0.16
	[-0.18, 1.07]	[-0.81, 0.56]	[-0.45, 0.89]	[-0.26, 1.05]	[0.03, 1.37]	[-0.73, 0.62]	[-0.44, 0.85]	[-0.86, 0.48]	[-0.72, 0.63]	[-0.51, 0.83]
Female	0.00	0.01	0.26	0.14	0.17	0.00	-0.15	-0.03	0.13	0.17
	[-0.26, 0.27]	[-0.28, 0.30]	[-0.03, 0.55]	[-0.14, 0.42]	[-0.12, 0.45]	[-0.29, 0.29]	[-0.43, 0.12]	[-0.32, 0.25]	[-0.16, 0.42]	[-0.11, 0.46]
R-Squared	0.15	-0.03	0.02	0.06	0.02	0.00	0.09	0.01	0.01	0.01
Observations	210	210	210	210	210	210	210	210	210	210

Standardized regression coefficients and 95% confidence intervals are reported for continuous variables. Coefficients on the binary variables are shaded gray and report the standard deviation change in the outcome by moving from 0 to 1 on the binary variable. Cohort fixed effects are included for each model. Adjusted R-squared is presented for each model.

*p < .05.

**p < .01.

***p < .001.

Some admissions decisions are made according to GRE percentiles, so all analysis were repeated with GRE percentiles and showed qualitatively similar results as with the GRE raw scores.

## Discussion

This analysis is designed to assist admissions committees who are responsible for evaluating candidates for positions in biomedical research graduate programs. The overall result of this study is that there is little objective information in the application to reliably identify future outstanding performers in research.

Data from Vanderbilt Medical School’s IGP reveal that few of the currently used objective criteria for admission demonstrate high levels of predictive validity for measures of progress in the program or research productivity. Importantly, the GRE provides no insight into such important graduate education measures as passing the qualifying exam, graduating with a Ph.D., time to defense, number of presentations, number of first author publications, or winning an individual grant or fellowship.

When examining classroom performance, GRE Quantitative and Verbal scores moderately predict first semester grades, and the GRE Verbal subtest minimally predicts graduate GPAs after accounting for other observable components of the applicant. The relationship between GRE scores and graduate school grades could be due to the GRE exam’s measuring characteristics such as test taking skills, attention, time management, stress management, test question comprehension, and reviewing one’s work. These skills likely overlap with the ability to succeed on graduate course exams and problem sets, and differ from the critical thinking, experimental design, and writing skills needed for the qualifying exam, research productivity and other measures of graduate school success. ETS [7] and Kuncel [5] argue that the GRE assesses cognitive skills and academic knowledge that relate to graduate school research ability, however, our results do not support such theories.” We also note that didactic coursework takes place in the first two years of graduate school whereas the other measures are captured more than two years after a student completed the GRE. The less time that passes between measures, the stronger the relationship [26, 27].

Interestingly, the GRE does moderately predict some elements in faculty evaluations of recently graduated students, which often occur over six years after the completion of the GRE. The most consistent pattern was found among GRE Verbal scores, which moderately predict faculty ratings of how well students handle classwork and minimally predict keeping up with literature, supporting our earlier finding that the GRE predicts success in the classroom. GRE Verbal scores also minimally predict writing ability, a highly verbal skill, yet writing ability does not appear to translate to the number of published first-author papers or a successful dissertation defense. Faculty had access to students’ GRE scores, which could have biased their responses, however, GRE Quantitative scores did not predict any elements of the evaluation, suggesting that faculty are not using this information when assessing their students. GRE subtests did not predict drive, experimental design, output, trajectory, or faculty evaluations of overall productivity as a scientist, suggesting that the GRE is more closely aligned with classroom behavior than laboratory performance.

Coursework is a traditional component of graduate education as currently performed in the United States. However, didactic courses are simply useful preparatory steps along the way to more important aspects of research training, namely the development of creative skills and technical abilities, time to degree, and productivity in terms of written and published materials. Thus, to the question of whether GRE scores can help guide us to select individuals with these research-specific skills: the answer is that they do not.

Variables other than the GRE are better predictors of graduate student success. Undergraduate GPA is a stronger predictor of graduate GPA, first semester grades, and graduating with a Ph.D. than GRE scores. Moreover, all of the objective admissions criteria explain only a small portion of the variance observed in most outcomes, meaning the admission criteria are missing many critical components of students’ success. Some of those components may be gleaned from letters and the personal statement. A new study reveals that letters of recommendation predict first author publication counts [28]. Admissions committees might consider placing more weight on these criteria instead of on GRE scores, depending on the outcome measures deemed most important in their program.

Underrepresented minorities were more likely to obtain individual grants or fellowships, possibly due to a number of diversity fellowships that are only available to this population. Although underrepresented minorities had lower first semester grades, GPAs, and lower odds of graduating with a Ph.D., with academic and social supports, undergraduate science and engineering programs have been shown to improve minority graduation rates [29,30], a finding supported by preliminary data on our own graduate population. On most other measures, underrepresented minority status had no meaningful impact, revealing that neither underrepresented minority status nor GRE scores predicted who would be a productive scientist. The key take-away is to move away from using GRE scores in admissions decisions, as they have little value in predicting success in the biomedical research enterprise, and may in fact run counter to the goal of diversifying the biomedical research workforce.

Importantly, these findings are limited to only the students admitted to and enrolled in Vanderbilt’s IGP and do not include all applicants since we cannot observe outcomes on students who did not enroll. This is a common source of bias that exists in nearly all predictive validity studies of standardized tests used in admission processes. Since we do not observe the outcomes of students who did not enroll, we must assume the predictive validity of GRE scores on matriculants is similar to what it would have been for applicants who were not admitted or did not choose to enroll. Furthermore, our study does not include a random sample of the entire range of GRE scores. The students entering graduate school at Vanderbilt have been chosen by traditional criteria that rely on conventional wisdom that there is decreased performance below a certain GRE score. As such, the sample of students has GRE Verbal and Quantitative scores roughly 100 points higher than the national average for each subtest [31].

In summary, our recommendations are not radically different from those of ETS who urge that GRE test scores not be the sole arbiter of admissions to graduate programs. However, we would go one step further, at least for applications to graduate school in biomedical sciences research, and advise that these standardized tests are unlikely to provide the important information needed to determine success in graduate school. For Vanderbilt’s IGP, GRE scores are mainly valid as predictors of performance in didactic coursework, but not for any other important measures of success in graduate school such as graduating with a Ph.D. or research productivity. Importantly, given the racial and socioeconomic differences in test performance, a strong reliance on GRE scores for admissions may negatively impact specific groups of students and could reduce the diversity of students in a program. The limited benefits of the GRE do not outweigh the potential costs of excluding minority and low socioeconomic status applicants.

## Supporting Information

S1 Supporting Information Details regarding the sample and additional results tables. (DOCX)
