# Supplementary material for: The Limitations of the GRE in Predicting Success in Biomedical Graduate School
Source: PLoS One. 2017 Jan 11;12(1):e0166742. doi: 10.1371/journal.pone.0166742 (PMC5226333; doi:10.1371/journal.pone.0166742)
Supplement: S1 Supporting Information — (DOCX) [file pone.0166742.s001.docx]

**Supporting Information**

**Sample**

From the initial 683 students who matriculated into the IGP from 2003 to 2011, 188 students were missing one or more independent variables: 122 did not submit a GRE Writing score (which was recommended but not required on their applications), 65 were excluded for not having undergraduate college admissions rates in the U.S. Department of Education Integrated Postsecondary Education Data System, and one student’s undergraduate GPA was not on a 4.0 scale. This left a subsample of 495 students who had values for all of the independent variables. T-tests revealed that this subset differed slightly from the students for whom we do not have complete data, in regard to independent variables. The 495 student subsample has higher GRE Verbal scores (M: 554.26 vs. 516.98, t(632)=4.32, p<.001), higher GRE Writing scores (M: 4.62 vs. 4.04, t(559)=6.54, p<.001), higher undergraduate GPAs (M: 3.54 vs. 3.39, t(608)=4.37, p<.001), a lower proportion with a prior advanced degree (M: 0.05 vs. 0.30, t(681) = -9.96, p<.001), a lower proportion with underrepresented minority status (M: 0.12 vs. 0.21, t(681)= -2.79, p<.01), and a lower proportion of international students (M: 0.05 vs. 0.30, t(681)= -9.96, p<.001). The differences between groups may be explained by the lower proportion of international students included in the 495 subsample. International students who attended international undergraduate institutions were excluded for not having undergraduate college admissions rates in the U.S. Department of Education Integrated Postsecondary Education Data System. The lower proportion of underrepresented minorities in the subsample is driven by the reduced number of minority students who submitted GRE Writing scores. No group differences were found among the dependent variables.

**Results**

The following tables show regression coefficients and R^2^ values for the relationship between admissions criteria and Passing the Qualifying Exam (Table A), Time to Defense (Table B), Presentation Count (Table C), First Author Publication Count (Table D), and obtaining an Individual Grant or Fellowship (Table E). None of the coefficients of interest on GRE scores are statistically significant suggesting that these objective admissions criteria are not useful in predicting any of these outcomes.

**Table A. The predictive power of GRE scores and other admissions criteria on Passing the Qualifying Exam.**

|  | (1) | (2) | (3) | (4) | (5) | (6) | (7) | (8) | (9) |
| --- | --- | --- | --- | --- | --- | --- | --- | --- | --- |
| GRE Quantitative | 0.03* | 0.03 | 0.03 | 0.03 | 0.03 | 0.03 | 0.03 | 0.02 | 0.02 |
|  | [0.00, 0.06] | [0.00, 0.06] | [-0.01, 0.06] | [-0.01, 0.06] | [-0.01, 0.06] | [-0.01, 0.06] | [-0.01, 0.05] | [-0.01, 0.06] | [-0.01, 0.06] |
| GRE Verbal |  | 0.01 | 0.00 | 0.00 | 0.00 | 0.00 | 0.00 | 0.01 | 0.01 |
|  |  | [-0.03, 0.04] | [-0.03, 0.04] | [-0.03, 0.04] | [-0.03, 0.04] | [-0.03, 0.04] | [-0.03, 0.04] | [-0.03, 0.04] | [-0.03, 0.04] |
| GRE Writing |  |  | 0.00 | 0.00 | 0.00 | 0.00 | 0.00 | 0.01 | 0.01 |
|  |  |  | [-0.03, 0.04] | [-0.03, 0.04] | [-0.03, 0.04] | [-0.03, 0.04] | [-0.03, 0.04] | [-0.03, 0.04] | [-0.03, 0.04] |
| Undergraduate GPA |  |  |  | 0.01 | 0.01 | 0.01 | 0.01 | 0.00 | 0.00 |
|  |  |  |  | [-0.02, 0.04] | [-0.03, 0.04] | [-0.03, 0.04] | [-0.03, 0.04] | [-0.03, 0.03] | [-0.03, 0.04] |
| Undergraduate Inst. Selectivity |  |  |  |  | 0.00 | 0.00 | 0.00 | 0.01 | 0.01 |
|  |  |  |  |  | [-0.03, 0.03] | [-0.03, 0.03] | [-0.03, 0.03] | [-0.03, 0.04] | [-0.03, 0.04] |
| Prior Advanced Degree |  |  |  |  |  | 0.01 | 0.01 | 0.01 | 0.01 |
|  |  |  |  |  |  | [-0.13, 0.15] | [-0.13, 0.15] | [-0.14, 0.15] | [-0.14, 0.15] |
| Underrepresented Minority |  |  |  |  |  |  | -0.02 | -0.03 | -0.03 |
|  |  |  |  |  |  |  | [-0.12, 0.07] | [-0.13, 0.06] | [-0.13, 0.06] |
| International |  |  |  |  |  |  |  | 0.08 | 0.08 |
|  |  |  |  |  |  |  |  | [-0.06, 0.23] | [-0.06, 0.23] |
| Female |  |  |  |  |  |  |  |  | 0.00 |
|  |  |  |  |  |  |  |  |  | [-0.06, 0.06] |
| Adjusted R-Squared | 0.02 | 0.02 | 0.02 | 0.02 | 0.01 | 0.01 | 0.01 | 0.01 | 0.01 |
| Observations | 495 | 495 | 495 | 495 | 495 | 495 | 495 | 495 | 495 |

Continuous independent variables were standardized prior to entering the models. Binary independent variables were not standardized and are shaded gray. Cohort fixed effects are included for each model. Regression coefficients and 95% confidence intervals are reported, along with adjusted R-squared for each model.

*p < .05. **p < .01. ***p < .001.

**Table B. The predictive power of GRE scores and other admissions criteria on Time to Defense.**

|  | (1) | (2) | (3) | (4) | (5) | (6) | (7) | (8) | (9) |
| --- | --- | --- | --- | --- | --- | --- | --- | --- | --- |
| GRE Quantitative | 0.00 | -0.04 | -0.03 | -0.02 | -0.02 | 0.00 | -0.01 | -0.01 | -0.02 |
|  | [-0.11, 0.10] | [-0.16, 0.07] | [-0.15, 0.09] | [-0.14, 0.10] | [-0.14, 0.10] | [-0.12, 0.12] | [-0.14, 0.12] | [-0.13, 0.12] | [-0.15, 0.10] |
| GRE Verbal |  | 0.10 | 0.12* | 0.13* | 0.13* | 0.12* | 0.12* | 0.12 | 0.11 |
|  |  | [-0.02, 0.22] | [0.00, 0.24] | [0.01, 0.25] | [0.01, 0.25] | [0.00, 0.24] | [0.00, 0.24] | [0.0, 0.24] | [-0.01, 0.24] |
| GRE Writing |  |  | -0.10 | -0.10 | -0.10 | -0.10 | -0.10 | -0.10 | -0.10 |
|  |  |  | [-0.22, 0.02] | [-0.21, 0.02] | [-0.21, 0.02] | [-0.21, 0.02] | [-0.22, 0.02] | [-0.22, 0.02] | [-0.22, 0.02] |
| Undergraduate GPA |  |  |  | -0.08 | -0.08 | -0.07 | -0.07 | -0.06 | -0.05 |
|  |  |  |  | [-0.19, 0.03] | [-0.20, 0.03] | [-0.18, 0.05] | [-0.18, 0.05] | [-0.18, 0.05] | [-0.17, 0.07] |
| Undergraduate Inst. Selectivity |  |  |  |  | 0.00 | 0.00 | 0.00 | 0.00 | 0.01 |
|  |  |  |  |  | [-0.11, 0.12] | [-0.11, 0.12] | [-0.12, 0.12] | [-0.12, 0.11] | [-0.13, 0.11] |
| Prior Advanced Degree |  |  |  |  |  | 0.36 | 0.37 | 0.38 | 0.37 |
|  |  |  |  |  |  | [-0.15, 0.87] | [-0.15, 0.88] | [-0.14, 0.90] | [-0.14, 0.90] |
| Underrepresented Minority |  |  |  |  |  |  | -0.11 | -0.08 | -0.08 |
|  |  |  |  |  |  |  | [-0.51, 0.29] | [-0.49, 0.32] | [-0.49, 0.32] |
| International |  |  |  |  |  |  |  | -0.15 | -0.16 |
|  |  |  |  |  |  |  |  | [-0.63, 0.33] | [-0.64, 0.31] |
| Female |  |  |  |  |  |  |  |  | -0.19 |
|  |  |  |  |  |  |  |  |  | [-0.41, 0.03] |
| Adjusted R-Squared | 0.07 | 0.08 | 0.08 | 0.09 | 0.08 | 0.09 | 0.08 | 0.08 | 0.09 |
| Observations | 318 | 318 | 318 | 318 | 318 | 318 | 318 | 318 | 318 |

The outcome is standardized. Continuous independent variables were standardized prior to entering the models. Binary independent variables were not standardized and are shaded gray. Cohort fixed effects are included for each model. Standardized regression coefficients and 95% confidence intervals are reported for continuous variables. Coefficients on the binary variables report the standard deviation change in the outcome by moving from 0 to 1 on the binary variable. Adjusted R-squared is presented for each model.

*p < .05. **p < .01. ***p < .001.

**Table C. The predictive power of GRE scores and other admissions criteria on Presentation Count.**

|  | (1) | (2) | (3) | (4) | (5) | (6) | (7) | (8) | (9) |
| --- | --- | --- | --- | --- | --- | --- | --- | --- | --- |
| GRE Quantitative | 0.06 | 0.04 | 0.03 | 0.03 | 0.03 | 0.04 | 0.04 | 0.03 | 0.04 |
|  | [-0.07, 0.18] | [-0.09, 0.18] | [-0.10, 0.17] | [-0.11, 0.16] | [-0.11, 0.16] | [-0.10, 0.18] | [-0.10, 0.18] | [-0.11, 0.18] | [-0.11, 0.18] |
| GRE Verbal |  | 0.01 | 0.01 | 0.01 | 0.01 | 0.01 | 0.01 | 0.03 | 0.03 |
|  |  | [-0.10, 0.16] | [-0.12, 0.15] | [-0.13, 0.15] | [-0.13, 0.15] | [-0.13, 0.15] | [-0.13, 0.15] | [-0.12, 0.17] | [-0.12, 0.17] |
| GRE Writing |  |  | 0.06 | 0.05 | 0.05 | 0.05 | 0.05 | 0.06 | 0.05 |
|  |  |  | [-0.08, 0.19] | [-0.08, 0.19] | [-0.09, 0.18] | [-0.09, 0.18] | [-0.09, 0.18] | [-0.08, 0.19] | [-0.08, 0.19] |
| Undergraduate GPA |  |  |  | 0.07 | 0.08 | 0.09 | 0.09 | 0.08 | 0.08 |
|  |  |  |  | [-0.05, 0.20] | [-0.05, 0.21] | [-0.04, 0.22] | [-0.04, 0.22] | [-0.05, 0.22] | [-0.05, 0.23] |
| Undergraduate Inst. Selectivity |  |  |  |  | -0.05 | -0.05 | -0.04 | -0.03 | -0.03 |
|  |  |  |  |  | [-0.18, 0.09] | [-0.18, 0.09] | [-0.18, 0.09] | [-0.17, 0.10] | [-0.17, 0.10] |
| Prior Advanced Degree |  |  |  |  |  | 0.30 | 0.30 | 0.26 | 0.26 |
|  |  |  |  |  |  | [-0.31, 0.90] | [-0.31, 0.90] | [-0.35, 0.87] | [-0.35, 0.87] |
| Underrepresented Minority |  |  |  |  |  |  | 0.05 | 0.00 | 0.00 |
|  |  |  |  |  |  |  | [-0.40, 0.50] | [-0.46, 0.46] | [-0.46, 0.46] |
| International |  |  |  |  |  |  |  | 0.39 | 0.40 |
|  |  |  |  |  |  |  |  | [-0.18, 0.97] | [-0.18, 0.97] |
| Female |  |  |  |  |  |  |  |  | 0.04 |
|  |  |  |  |  |  |  |  |  | [-0.22, 0.30] |
| Adjusted R-Squared | -0.02 | -0.02 | -0.02 | 0.02 | -0.03 | -0.03 | -0.03 | -0.03 | -0.03 |
| Observations | 271 | 271 | 271 | 271 | 271 | 271 | 271 | 271 | 271 |

The outcome is standardized. Continuous independent variables were standardized prior to entering the models. Binary independent variables were not standardized and are shaded gray. Cohort fixed effects are included for each model. Standardized regression coefficients and 95% confidence intervals are reported for continuous variables. Coefficients on the binary variables report the standard deviation change in the outcome by moving from 0 to 1 on the binary variable. Adjusted R-squared is presented for each model.

*p < .05. **p < .01. ***p < .001.

**Table D. The predictive power of GRE scores and other admissions criteria on First Author Publication Count.**

|  | (1) | (2) | (3) | (4) | (5) | (6) | (7) | (8) | (9) |
| --- | --- | --- | --- | --- | --- | --- | --- | --- | --- |
| GRE Quantitative | 0.04 | 0.02 | 0.02 | 0.02 | 0.02 | 0.02 | 0.04 | 0.03 | 0.02 |
|  | [-0.08, 0.16] | [-0.11, 0.15] | [-0.11, 0.15] | [-0.12, 0.15] | [-0.12, 0.15] | [-0.12, 0.15] | [-0.10, 0.18] | [-0.11, 0.18] | [-0.12, 0.16] |
| GRE Verbal |  | 0.05 | 0.05 | 0.05 | 0.05 | 0.05 | 0.05 | 0.06 | 0.05 |
|  |  | [-0.08, 0.19] | [-0.09, 0.19] | [-0.09, 0.19] | [-0.09, 0.18] | [-0.09, 0.18] | [-0.08, 0.19] | [-0.08, 0.20] | [-0.09, 0.19] |
| GRE Writing |  |  | 0.02 | 0.01 | 0.01 | 0.01 | 0.01 | 0.02 | 0.02 |
|  |  |  | [-0.12, 0.15] | [-0.12, 0.15] | [-0.13, 0.14] | [-0.13, 0.14] | [-0.12, 0.15] | [-0.12, 0.15] | [-0.12, 0.15] |
| Undergraduate GPA |  |  |  | 0.03 | 0.04 | 0.04 | 0.04 | 0.04 | 0.04 |
|  |  |  |  | [-0.10, 0.15] | [-0.09, 0.17] | [-0.09, 0.17] | [-0.09, 0.17] | [-0.09, 0.17] | [-0.09, 0.17] |
| Undergraduate Inst. Selectivity |  |  |  |  | -0.05 | -0.05 | -0.03 | -0.03 | -0.03 |
|  |  |  |  |  | [-0.18, 0.08] | [-0.18, 0.08] | [-0.17, 0.10] | [-0.17, 0.10] | [-0.17, 0.10] |
| Prior Advanced Degree |  |  |  |  |  | 0.08 | 0.09 | 0.07 | 0.08 |
|  |  |  |  |  |  | [-0.52, 0.68] | [-0.51, 0.68] | [-0.53, 0.68] | [-0.52, 0.68] |
| Underrepresented Minority |  |  |  |  |  |  | 0.28 | 0.27 | 0.27 |
|  |  |  |  |  |  |  | [-0.16, 0.73] | [-0.19, 0.72] | [-0.18, 0.73] |
| International |  |  |  |  |  |  |  | 0.13 | 0.12 |
|  |  |  |  |  |  |  |  | [-0.44. 0.70] | [-0.45. 0.68] |
| Female |  |  |  |  |  |  |  |  | -0.15 |
|  |  |  |  |  |  |  |  |  | [-0.41, 0.11] |
| Adjusted R-Squared | 0.00 | 0.00 | 0.00 | 0.00 | -0.01 | -0.01 | -0.01 | -0.01 | -0.01 |
| Observations | 271 | 271 | 271 | 271 | 271 | 271 | 271 | 271 | 271 |

The outcome is standardized. Continuous independent variables were standardized prior to entering the models. Binary independent variables were not standardized and are shaded gray. Cohort fixed effects are included for each model. Standardized regression coefficients and 95% confidence intervals are reported for continuous variables. Coefficients on the binary variables report the standard deviation change in the outcome by moving from 0 to 1 on the binary variable. Adjusted R-squared is presented for each model.

*p < .05. **p < .01. ***p < .001.

**Table E. The predictive power of GRE scores and other admissions criteria on obtaining an Individual Grant or Fellowship.**

|  | (1) | (2) | (3) | (4) | (5) | (6) | (7) | (8) | (9) |
| --- | --- | --- | --- | --- | --- | --- | --- | --- | --- |
| GRE Quantitative | 0.00 | 0.00 | *-0.01* | -0.01 | -0.02 | -0.01 | 0.00 | 0.00 | 0.01 |
|  | [-0.06, 0.06] | [-0.06, 0.06] | *[-0.07, 0.06]* | [-0.07, 0.05] | [-0.08, 0.05] | [-0.08, 0.05] | [-0.06, 0.07] | [-0.06, 0.07] | [-0.06, 0.08] |
| GRE Verbal |  | 0.00 | *-0.02* | -0.02 | -0.02 | -0.02 | -0.01 | -0.01 | -0.01 |
|  |  | [-0.07, 0.06] | *[-0.08, 0.05]* | [-0.08, 0.05] | [-0.09, 0.05] | [-0.09, 0.05] | [-0.08, 0.05] | [-0.08, 0.05] | [-0.07, 0.06] |
| GRE Writing |  |  | *0.05* | 0.05 | 0.04 | 0.04 | 0.05 | 0.05 | 0.05 |
|  |  |  | *[-0.01, 0.12]* | [-0.01, 0.12] | [-0.02, 0.11] | [-0.02, 0.11] | [-0.01, 0.11] | [-0.01, 0.12] | [-0.01, 0.11] |
| Undergraduate GPA |  |  |  | 0.02 | 0.03 | 0.03 | 0.03 | 0.03 | 0.02 |
|  |  |  |  | [-0.04, 0.08] | [-0.03, 0.09] | [-0.03, 0.10] | [-0.03, 0.09] | [-0.04, 0.09] | [-0.04, 0.09] |
| Undergraduate Inst. Selectivity |  |  |  |  | -0.06 | -0.06 | -0.04 | -0.04 | -0.04 |
|  |  |  |  |  | [-0.12, 0.01] | [-0.12, 0.01] | [-0.11, 0.02] | [-0.10, 0.02] | [-0.10, 0.02] |
| Prior Advanced Degree |  |  |  |  |  | 0.02 | 0.02 | 0.02 | 0.01 |
|  |  |  |  |  |  | [-0.26, 0.31] | [-0.26, 0.31] | [-0.27, 0.30] | [-0.27, 0.30] |
| Underrepresented Minority |  |  |  |  |  |  | 0.26* | 0.25* | 0.25* |
|  |  |  |  |  |  |  | [0.05, 0.48] | [0.04, 0.47] | [0.04, 0.47] |
| International |  |  |  |  |  |  |  | 0.09 | 0.10 |
|  |  |  |  |  |  |  |  | [-0.18, 0.36] | [-0.17, 0.37] |
| Female |  |  |  |  |  |  |  |  | 0.07 |
|  |  |  |  |  |  |  |  |  | [-0.06, 0.19] |
| Adjusted R-Squared | 0.02 | 0.01 | 0.02 | 0.02 | 0.03 | 0.02 | 0.04 | 0.04 | 0.04 |
| Observations | 271 | 271 | 271 | 271 | 271 | 271 | 271 | 271 | 271 |

Continuous independent variables were standardized prior to entering the models. Binary independent variables were not standardized and are shaded gray. Cohort fixed effects are included for each model. Regression coefficients and 95% confidence intervals are reported, along with adjusted R-squared for each model.

*p < .05. **p < .01. ***p < .001.
